# A Clinical Perspective on Multidimensional Assessment of Clinical High-Risk States for Psychosis Beyond Transition Prediction

**DOI:** 10.3390/jcm15207955

**Published:** 2026-10-14

**Authors:** Octavian Vasiliu, Ileana Marinescu, Andrei G. Mangalagiu, Bogdan M. Petrescu, Adela M. Ciobanu

**Affiliations:** 1 Discipline of Psychiatry II, Neurosciences Department, Faculty of Medicine, “Carol Davila” University of Medicine and Pharmacy, 020021 Bucharest, Romania; octavian.vasiliu@umfcd.ro (O.V.); bogdan.petrescu@umfcd.ro (B.M.P.); 2 Clinical Department of Psychiatry, Central Military Emergency University Hospital “Dr. Carol Davila”, 010825 Bucharest, Romania; 3 Department of Psychiatry, Faculty of Medicine, University of Medicine and Pharmacy of Craiova, 200349 Craiova, Romania; 4 Psychiatry Clinic, Craiova Clinical Hospital for Neuropsychiatry, 200473 Craiova, Romania; 5 Discipline of Psychiatry I, Neurosciences Department, Faculty of Medicine, “Carol Davila” University of Medicine and Pharmacy, 020021 Bucharest, Romania; adela.ciobanu@umfcd.ro; 6 9th Department of Psychiatry, “Prof. Dr. Al. Obregia” Clinical Hospital of Psychiatry, 041914 Bucharest, Romania

**Keywords:** psychosis, clinical high risk, early intervention, clinical assessment, psychosocial functioning, longitudinal monitoring

## Abstract

**Background:** Clinical high-risk states for psychosis (CHR-P) are associated with heterogeneous symptomatic and functional outcomes, only one of which is transition to psychosis. **Objective:** To propose a structured approach to current care needs and longitudinal clinical formulation alongside established syndromic assessment. **Methods:** This perspective integrates a targeted, non-systematic appraisal of instrument studies, evidence syntheses, longitudinal cohorts, and guidance with an explicitly author-proposed assessment architecture. Supplementary PubMed searches conducted during revision have been reported separately from the original search. **Results:** The framework retains validated CHR-P interviews as the syndromic anchor and organizes symptoms, functioning, cognition, comorbidity, developmental context, and longitudinal change into descriptive clinical profiles. A minimal clinical dataset is distinguished from optional structured assessments and investigational biological or digital measures. Descriptive profiles document source measures, current burden, change, and uncertainty without numerical weighting. A staged validation program prioritizes reproducibility, feasibility, and functioning, with transition assessed separately. **Conclusions:** The current framework offers a candidate structure for documenting needs and discussing care priorities; it does not establish prognostic accuracy or treatment benefit. Prospective evaluation is required before profile-based decisions based on this interpretative model can be recommended. A clinically useful approach to CHR-P research may need to move beyond reductionist conversion/non-conversion predictive models toward more calibrated, externally validated paradigms capable of predicting multiple clinically meaningful trajectories.

## 1. Introduction

The concept of a psychosis risk state developed from multiple historical attempts to operationalize the difficult-to-circumscribe, phenomenologically challenging, and therapeutically puzzling, but also functionally provocative, prodromal period of schizophrenia. The history of the high-risk for psychosis concept can be traced back to the founding fathers of modern psychiatry, Emil Kraepelin and Eugen Bleuler, who noted that full-blown psychosis was frequently preceded by nonspecific manifestations, such as social withdrawal, reduced functioning, affective changes, bizarre behavior, and discrete disturbances of thinking processes [1,2,3,4]. However, these early accounts were retrospective and purely descriptive, thus suffering from the caveats of early scientific methodology, resulting in the prodrome of schizophrenia being recognized with certainty only after the psychosis had already developed. Therefore, the term prodrome originally referred to the period extending from the first detectable change from premorbid functioning to the onset of frank psychosis, rather than to a prospectively identifiable clinical syndrome [1,2]. During the second half of the twentieth century, retrospective studies attempted to characterize this stage in a more systematic manner, and their results highlighted the existence of social isolation, deterioration in occupational or academic performance, reduced motivation, sleep disturbance, anxiety, depressive symptoms, suspiciousness, impaired concentration, and attenuated perceptual or ideational abnormalities [1,2]. The American Psychiatric Association’s Diagnostic and Statistical Manual of Mental Disorders, Third Edition, Revised (DSM-III-R) marked an important step toward acknowledging this concept by operationalizing it through a list of nine prodromal or residual manifestations of schizophrenia [5]. Nevertheless, these features were of limited specificity, because many also occurred in mood, anxiety, personality, substance-related, and neurodevelopmental disorders, thus presenting little diagnostic value. Due to this conceptual imprecision, the fourth edition of the DSM did not move forward and failed to establish a separately diagnosable prodromal syndrome of schizophrenia [6].

A distinct European line of research emerged from Gerd Huber’s concept of basic symptoms, and this direction proved itself worthy of further investigation by clinicians of subsequent generations [7,8]. These symptoms were defined by Huber as discrete, subjectively experienced disturbances across a variety of domains, starting from cognition to perception, and from language or affect to volition and motor functioning, but generally without affecting reality testing [7]. The Cologne Early Recognition study later showed that selected basic cognitive-perceptual symptoms could precede first-episode psychosis (FEP) by several years and have clinically meaningful predictive value, thereby linking a theoretical construct to practical value [7]. This line of exploration produced several operational criteria, including basic cognitive-perceptual symptoms and cognitive dysfunctions, which were assessed using specific instruments such as the Schizophrenia Proneness Instrument (SPI) [7,9]. Unlike approaches centered on attenuated positive symptoms, Huber’s model targeted earlier, self-perceived changes that may precede externally observable psychotic phenomena in the schizophrenia spectrum disorders (SSD).

Yung et al. introduced a series of significant constructs, i.e., the at-risk mental state (ARMS) and ultra-high-risk of psychosis (UHR-P), distinguishing brief limited intermittent psychotic symptoms (BLIPS), attenuated positive symptoms, and vulnerability states characterized by trait risk combined with functional impairment [10]. The Structured Interview for Psychosis-Risk Syndromes/Scale of Psychosis-Risk Symptoms (SIPS/SOPS) direction of investigation subsequently operationalized these states and added dimensional ratings of positive, negative, disorganization, and general psychopathology [11,12]. Comprehensive Assessment of At-Risk Mental States (CAARMS) was developed in parallel as a comprehensive instrument for ARMS by Yung et al. (2005) within the Melbourne early-psychosis research group [13]. These advances have made high-risk for psychosis ascertainment more clinically and scientifically reproducible, creating a scientific framework for research in this field, but they still have not solved the core interpretive problem. However, large longitudinal initiatives, including the North American Prodrome Longitudinal Study, demonstrated that combining attenuated psychotic symptoms with functional decline, suspiciousness, cognitive disturbances, and familial vulnerability could improve individualized risk estimation, and supported the utility of using validated instruments in the domain of high risk for psychosis (in this particular case, SIPS was administered to the target population of the study, *N* = 291 participants) [14].

During the 2000s and 2010s, the terminology gradually shifted from prodromal schizophrenia to clinical high risk for psychosis (CHR-P). The reasons for this paradigmatic change are multiple, the most important being that only a minority of people meeting high-risk criteria finally developed a psychotic disorder, as reflected by a landmark meta-analysis (*n* = 27 studies, *N* = 2502 patients) estimating transition risks of approximately 18% at 6 months, 22% at 1 year, 29% at 2 years, and 36% at 3 years [15]. Also, schizophrenia was not the only possible outcome of the transitioning phase, but affective or other psychotic disorders were possible, too, with many non-transitioning individuals continuing to experience attenuated symptoms, comorbid depression or anxiety, and impaired social or occupational functioning [16,17]. The CHR-P construct therefore requires a cautious interpretation as a heterogeneous clinical state encompassing multiple possible trajectories of evolution, rather than as an inescapable stage in the progression towards an SSD.

The inclusion of attenuated psychosis syndrome (APS) as a formal diagnosis in DSM-5 provoked considerable debate regarding its predictive validity, false-positive identification, stigma, and potentially unnecessary antipsychotic treatment. As a compromise formula, APS was included solely in Section III of DSM-5 and retained in DSM-5-TR, among conditions requiring further study rather than an established diagnostic category [18,19,20]. This critical decision reinforced the distinction between a risk syndrome and an already established psychotic disorder, preserving the border between the two categories both at the conceptual and practical levels.

**Figure 1:**
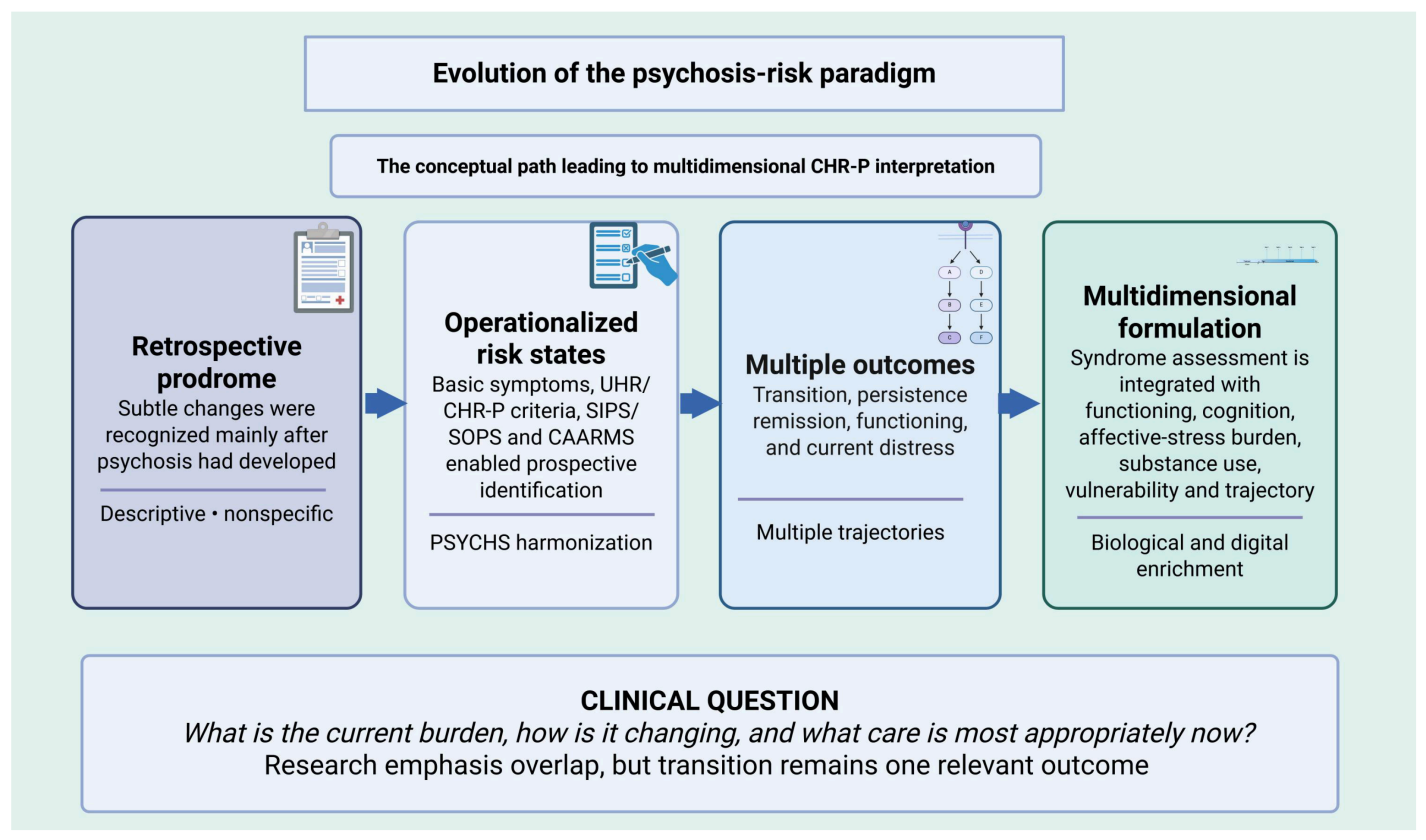
**Conceptual evolution of the psychosis-risk paradigm.** The stages summarize changing research emphases instead of mutually exclusive historical periods. CHR-P = Clinical high risk for psychosis; UHR = ultra-high risk; SIPS = Structured Interview for Psychosis-Risk Syndromes; SOPS = Scale of Psychosis-Risk Symptoms; CAARMS = Comprehensive Assessment of At-Risk Mental States; PSYCHS = Positive SYmptoms and Diagnostic Criteria for the CAARMS Harmonized with the SIPS. Created in BioRender. Vasiliu, O. (2026). https://BioRender.com/7fc5ng5. (accessed on 5 September 2026).

More recently, the field of CHR-P has moved from binary transition prediction toward multidimensional and longitudinal risk formulation (Figure 1). Biological, neuroimaging, electrophysiological, and digitally derived measures are increasingly regarded as potential enrichment layers rather than independent diagnostic gatekeepers. The Positive SYmptoms and Diagnostic Criteria for the CAARMS Harmonized with the SIPS (PSYCHS) was introduced in 2024 to reduce discrepancies between the two major assessment traditions, i.e., the CAARMS and SIPS traditions [21,22,23]. PSYCHS achieved full harmonization of attenuated positive-symptom ratings and psychosis criteria, although some differences between the respective CHR-P diagnostic algorithms remain [21,22,23].

Therefore, CHR-P should be considered a risk state for psychosis rather than a specific prodrome of schizophrenia, with heterogeneous criteria in baseline burden, mechanisms, trajectories, and outcomes that prevent the formulation of clear predictions [24,25,26,27,28]. Supporting this perspective, the results of an updated meta-analysis (*n* = 130 studies, *N* = 9222 individuals at CHR-P) showed the rate of transition to psychosis within three years is approximately 25%, with risk continuing to accumulate over longer follow-up [25]. Absolute risks vary across settings and syndromic subgroups, with BLIPS generally carrying greater transition risk than attenuated symptoms or genetic-risk/deterioration criteria.

Transition alone does not capture the outcomes that matter to patients. Persistent symptoms, functional impairment, and incomplete remission affect many individuals who do not transition [29,30]. A recent quality-of-life meta-analysis also documented substantial burden alongside variable improvement over follow-up [31]. Assessment must therefore address both possible psychosis onset and the person’s current difficulties, goals, and changing care needs.

This literature-informed perspective proposes an explicit documentation structure for separating current clinical burden, observed longitudinal change, and uncertain prognostic interpretation. Its contribution is the organization and communication of established domains, rather than the discovery of new predictors or the demonstration of incremental predictive accuracy. The objectives are to define a feasible minimum dataset, describe provisional clinical profiles without a composite score, and specify how reproducibility, clinical usefulness, and any later prognostic applications should be evaluated.

## 2. Methods and Scope of the Conceptual Synthesis

The current author-led clinical perspective is informed by a targeted literature appraisal and iterative clinical integration, aimed at identifying the most relevant methods for guiding clinical care for these individuals. A focused PubMed search was conducted through 31 August 2026 to retrieve relevant theoretical or practical data to support this initiative. Search strategies combined terms for clinical high risk, ultra-high risk, psychosis risk, SIPS/SOPS, CAARMS, PSYCHS, transition, functioning, cognition, social cognition, negative symptoms, comorbidity, trauma/stress, suicidality, stigma, neuroimaging, electrophysiology, inflammation, genetics, risk prediction, digital phenotyping, and preventive intervention. The search was focused on seminal instrument papers, meta-analyses, systematic reviews, large multisite studies, and recent harmonization or validation initiatives. No language or time limits were applied (except for the end date previously mentioned). Because the manuscript is not conceived as a systematic review, no PRISMA flow diagram, exhaustive search claim, formal risk-of-bias assessment, or quantitative synthesis was undertaken.

To improve transparency and currency, we ran three supplementary PubMed searches on 27 September 2026, keeping 31 August 2026 as the publication-date cutoff. Appendix A provides the exact queries, retrieval counts, and selection approach. Retrieved titles were inspected, and abstracts and selected full texts directly relevant to assessment, outcomes, clinical utility, or reviewer-raised issues were examined purposively. Seminal instrument papers and current official guidance were retained separately. Priority was given to evidence syntheses, large or multisite cohorts, instrument-development studies, and clinical guidance; small or non-CHR-P studies were used only to illustrate explicitly qualified hypotheses. No exhaustive screening claim, duplicate independent selection, formal risk-of-bias rating, or quantitative synthesis is made.

### How Domains and Tiers Were Selected

Domain selection considered five questions: (1) relevance to current needs or safety; (2) availability of validated measurement; (3) consistency and directness of CHR-P evidence; (4) feasibility and acceptability; and (5) evidence of added prognostic or decision value beyond ordinary assessment. These considerations were applied qualitatively, and no numerical weighting, formal evidence grade, or consensus vote was used. Therefore, the hierarchy is evidence-informed, and no assumption of quantitative weighting is implied. Clinical actionability and feasibility justify Tier 1 even when transition prediction is weak; a statistical association alone does not justify routine testing.

Tier 1 comprises the proposed minimum clinical dataset in Table 1, while Tier 2 includes optional standardized cognitive or other symptom assessments selected for a defined clinical question; Tier 3 refers to investigational biological and digital measures. The particular allocation is an author-proposed synthesis of clinical purpose and feasibility, not an empirically established ranking. Medical investigations undertaken for a differential diagnosis are outside this hierarchy and are available whenever clinically indicated.

## 3. The Syndromic Anchor: Necessary, but Not Sufficient

### 3.1. SIPS/SOPS, CAARMS, and PSYCHS

SIPS/SOPS was explicitly designed to determine the presence or absence of psychosis and psychosis-risk syndromes while measuring symptom severity over time [11,12]. This makes it especially important for clinical research because its dimensional structure is clinically valuable, with negative symptoms, functional deterioration, dysphoria, sleep disturbance, and impaired tolerance of stress captured alongside attenuated positive symptoms [11,12]. Also, the CAARMS is an essential tool in the research of CHR-P, since it similarly operationalizes ARMS and benefits from significant predictive validity in help-seeking populations [13].

SIPS and CAARMS differ in some thresholds, functional criteria, and diagnostic algorithms [22]. PSYCHS harmonizes positive-symptom assessment and psychosis criteria while allowing both classifications to be derived [23]. Instrument choice should depend on training, age and language appropriateness, and service infrastructure; this perspective does not establish the superiority of one interview for routine care. European Psychiatric Association guidance supports structured assessment in specialized pathways and recognizes basic-symptom assessment with the SPI-A or SPI-CY where indicated [32].

### 3.2. Fallacies of the Binary Perspective in the Domain of CHR-P

Psychometric assessment enriches risk relative to ordinary help-seeking samples, but the predictive accuracy of such an approach is far from certain [27]. The field’s own risk calculators highlight the fact that transition to psychosis is influenced by potential combinations of symptom severity, social functioning, cognition, age, stress, and family variables rather than by attenuated positive symptoms alone [33]. Poor social functioning, disorganized communication, and deficits in verbal learning and processing speed have repeatedly emerged as informative candidates in clinical research [33,34]. Also, negative symptoms are common, persistent, and associated with both conversion and functional burden, besides being extremely difficult to treat, therefore posing important challenges for clinicians [35]. These findings support multidimensional evaluation but do not validate the present profiles.

This heterogeneity is clinically provocative since two individuals with similar positive-symptom ratings may differ profoundly, e.g., one of them may have a rapid functional decline and progress toward thought disorganization, while the other may have stable attenuated psychopathological phenomena arising on a background of anxiety, trauma, or neurodevelopmental vulnerability. Therefore, treating both individuals as equivalent because they meet the same CHR-P threshold risks both overmedicalization and undertreatment, with negative prognostic outcomes.

A 2026 meta-analysis of 14 studies and 4477 help-seekers reported transition in 14.7% of CHR-P-positive versus 1.6% of CHR-P-negative participants over the included follow-up periods [36]. The OASIS1000 study additionally examined hospitalization, treatment exposure, and suicide [37], while long-term NAPLS follow-up documented persistent symptoms [38]. These studies show why syndromic ascertainment and broader outcome assessment complement each other.

## 4. A Multidomain Conceptualization of CHR-P

Functioning and social-role decline must be treated as a core interpretive domain, since social and role functioning make a major contribution to individualized prediction [33,34], baseline functioning anticipates remission from the UHR state [39], and persistent functional impairment is common even without psychosis transition [29,30,31,40]. A practical assessment should therefore capture both the current level and the recent direction of change, using the Social and Occupational Functioning Assessment Scale (SOFAS) and, where feasible, the Global Functioning: Social (GF:S) and Global Functioning: Role (GF:R) scales [41,42,43]. The clinically relevant signal is considered not just a low score but a sustained or accelerated decline inconsistent with the person’s developmental context.

Cognition and social cognition are other core dimensions that require thorough evaluation in cases of CHR-P. Neurocognitive dysfunction is present across CHR-P samples and is more pronounced in several domains among those who later transition [44]. Cognitive difficulties are also important because they may impede education, work, communication, and engagement with care. The relevance of social-cognitive impairment to these needs is supported by a 2024 meta-analysis that found modestly worse baseline facial-emotion perception among later transitioners, although this does not establish a clinically usable threshold or incremental predictive value [45,46]. Routine assessment should include a brief clinical inquiry into cognitive difficulties, whereas performance testing is optional. The BACS and MCCB are examples selected based on the question, available norms, training, and resources [47,48,49]. Their original development in schizophrenia does not establish that either complete battery is necessary or optimal for every CHR-P presentation.

A recent umbrella review identified verbal-memory deficits among the more consistent cognitive transition correlates but emphasized the limited readiness of predictors for clinical application [50]. Thus, cognition may contribute to a separately validated prognostic model while also identifying present learning and support needs; neither use can be inferred solely from the other.

Negative symptoms and disorganization are especially important when evaluating their overall impact on CHR-P individuals’ daily functioning and quality of life: negative symptoms deserve explicit weighting because avolition, social withdrawal, reduced emotional expression, and role deterioration may dominate morbidity before frank psychosis onset, whereas disorganization adds relevant information, particularly when communication progressively deteriorates [35]. Any interpretative framework needs to distinguish primary symptoms from depression, medication effects, substance use, and developmental differences, due to prognostic, evaluation, and therapeutic implications. In the currently proposed framework, no numerical prognostic weight is assigned to these domains, and clinicians are encouraged to report them descriptively.

Affective burden, stress, trauma, and substance use are approached together due to their negative impact on prognosis, although they are not core manifestations in CHR-P. However, comorbid psychopathology is the rule rather than the exception in this population, and also in patients with SSD. A large meta-analysis (*n* = 312 studies) published in 2023 showed that approximately 75% of CHR-P participants had at least one comorbid mental disorder, outside the psychotic spectrum, most likely mood and anxiety disorders, followed by trauma-related disorders, personality disorders, and other pathologies [51]. Earlier work similarly showed that non-psychotic disorders are highly prevalent but do not map clearly onto subsequent psychosis transition [52]. Network analysis in adolescents with CHR-P (*N* = 111 participants, assessed with SIPS) suggests strong interconnections among negative, disorganization, anxiety, and depressive symptoms, while positive symptoms can be considered only in addition to the broader symptom network [53]. From a clinical perspective, affective manifestations require longitudinal and contextual interpretation because their presence alone cannot clarify whether they represent a comorbid disorder, a reaction to emerging psychotic experiences, or part of the evolving psychosis-spectrum phenotype [54].

Environmental factors also require careful consideration, and this perspective is supported by meta-analytic evidence (*n* = 44 studies, 54 risk factors assessed) linking CHR-P status with childhood trauma, adverse life events, perceived stress, tobacco exposure, unemployment, physical inactivity, affective comorbidity, and social disadvantage [55]. Cannabis and other substance use should be assessed in a structured manner because exposure can worsen psychotic experiences, impair functioning, and complicate the attribution of psychopathological manifestations, such as perceptual or thought disturbances [56]. Substance use is therefore a separate and potentially modifiable element, which is important because it may interact with pre-existing vulnerability factors, symptom expression, global functioning, treatment engagement, and subsequent psychosis risk [56]. Within the current framework, affective and stress-related variables influence both risk interpretation and immediate care needs, even when their independent predictive power for transition is modest.

Neurodevelopmental and familial vulnerabilities are important aspects of the CHR-P assessment, with developmental history helping to differentiate a progressive change from a longstanding neurodevelopmental phenotype; autism-spectrum traits, attention deficits, developmental delays, premorbid social difficulties, and learning problems may shape both the expression and interpretation of unusual experiences. Family history should be recorded routinely because it remains part of established CHR-P assessment, but treating it as a simple deterministic factor is not recommended. This cautious approach is reinforced by a systematic review and meta-analysis (*n* = 93 studies) published in 2026, which shows that familial high-risk variables are inconsistently reported and do not demonstrate a straightforward positive association with transition across the available CHR-P literature [57]. Based on the available evidence, family vulnerability should be considered contextual information within a broader trajectory, rather than treated as a dominant, independent predictive element.

## 5. The Potential Role of Biological and Digital Enrichment

The neurobiological literature provides a strong rationale for enrichment, but such markers are not yet ready for inclusion into a routine standalone classification. Neuroplasticity offers a larger biological interpretive framework for examining dynamic interactions among cognition, environmental exposures, stress, and clinical trajectories, rather than interpreting biological findings as fixed determinants of transition [58]. Structural MRI studies have identified subtle cortical thickness differences at baseline and accelerated thinning in some individuals who progress to psychosis [59,60,61]. Also, recent research suggests that adding cortical thinning to clinical risk models can improve discrimination in selected subgroups, particularly when symptom duration is shorter [62]. The addition of MRS/perfusion investigation was reported in studies that have also linked prefrontal GABA physiology to hippocampal perfusion in individuals with UHR [63]. At the same time, large-scale normative and heterogeneity analyses demonstrate that most of the CHR-P individuals lack a single reproducible neuroanatomical signature, underscoring the danger of interpreting a single MRI abnormality as a clinical diagnosis [59].

Glymphatic research offers a hypothesis linking sleep, astrocytic function, inflammation, and brain homeostasis. Barlattani et al. reported lower diffusion tensor imaging along the perivascular space (DTI-ALPS) indices in nine young adults hospitalized with acute psychosis than in twelve non-psychiatric controls [64]. This small cross-sectional study involved antipsychotic-treated patients and imperfectly matched controls; associations with sleep quality, thought disorganization, and cognition were not significant. DTI-ALPS is an indirect imaging measure rather than a direct test of clearance. Moreover, a subsequent study found lower indices in first-episode schizophrenia-spectrum illness but no significant CHR-P–control difference (23 CHR-P participants and 30 controls) [65]. These observations motivate research but, at least for now, neither establish a causal pathway nor support DTI-ALPS for CHR-P screening, prognosis, or treatment selection.

Electrophysiological markers are among the more replicated candidates because mismatch negativity (MMN) has been reported as impaired across the SSD and appears relevant to disease progression [66]; a focused systematic review and meta-analysis (*n* = 6 studies) found duration MMN to be particularly promising for forecasting transition, but P300 and sensory gating were also impaired in individuals with CHR-P [67]. Other physiological measures, including antisaccade performance, show smaller abnormalities in UHR than in FEP and are better considered endophenotypic or enrichment signals than having diagnostic value [68].

Research blood panels, polygenic scores, and experimental fluid markers belong to Tier 3. NAPLS blood classifiers [69] and meta-analytic inflammatory findings, including IL-6 [70], support investigation but do not establish a clinically usable individual threshold. Polygenic scores require ancestry-aware external validation and calibration [71]; inconsistent familial-risk findings should not themselves be treated as a test of polygenic-score performance [57]. Endocannabinoid findings remain exploratory [72]. Ordinary laboratory tests, EEG, MRI, or cerebrospinal fluid examination requested for a specific differential diagnosis follow standard clinical indications independently of the enrichment hierarchy.

**Figure 2:**
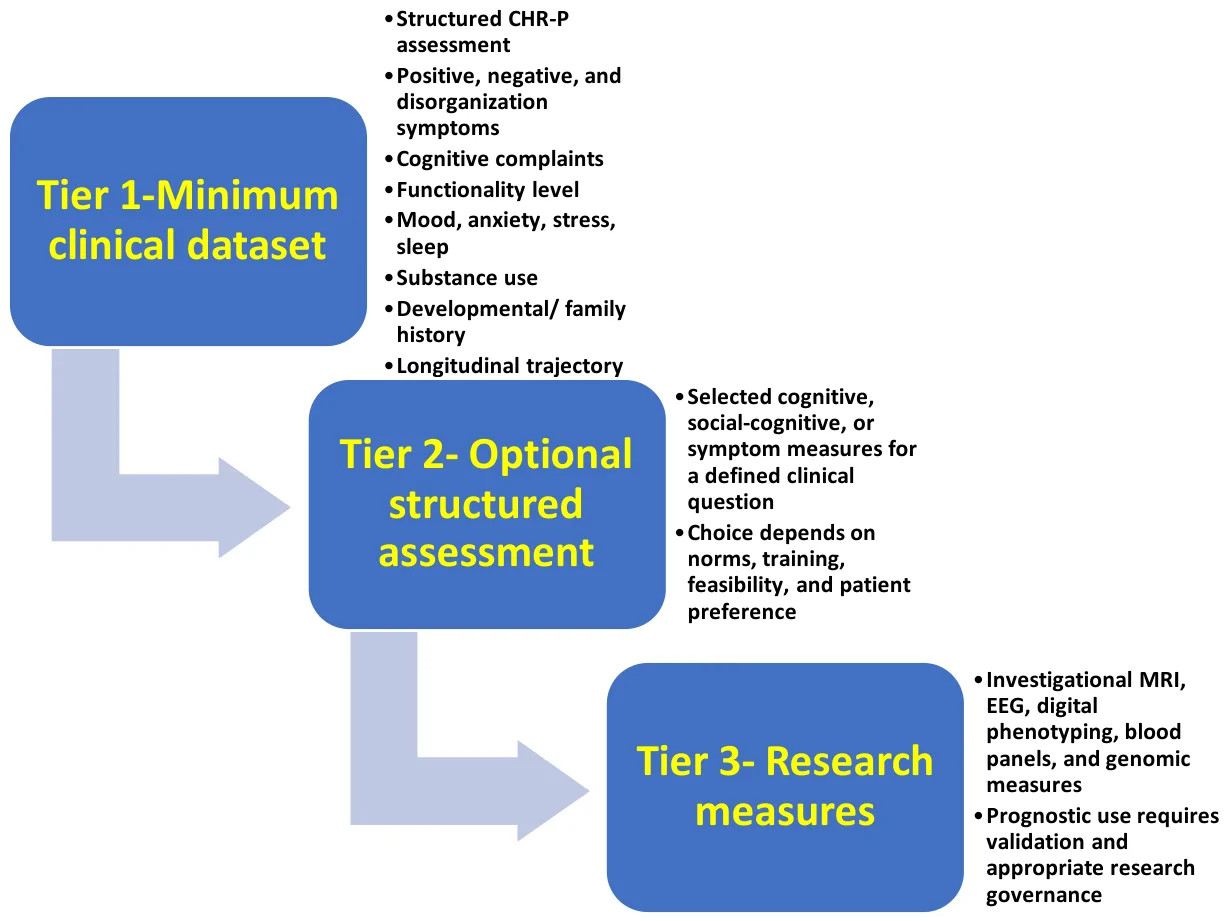
**Proposed assessment tiers in CHR-P according to clinical purpose and feasibility.** Tier 1 is the proposed minimum clinical dataset. Tier 2 contains optional structured assessments for a defined clinical question. Tier 3 contains investigational measures requiring appropriate research governance. Clinically indicated medical investigations remain available at any tier. The tiers are not mandatory sequential steps, treatment levels, or empirically derived evidence grades. CHR-P, clinical high risk for psychosis; MRI, magnetic resonance imaging; EEG, electroencephalography.

Digital phenotyping may be especially suited to a trajectory-centered framework because it can capture changes in sleep, mobility, communication, activity, and social rhythm between clinic monitoring visits. Recent CHR-P research has associated smartphone social-behavior measures with illness-progression risk scores [73], while the digital component of AMP SCZ is prospectively collecting active and passive smartphone data and actigraphy within an internationally harmonized cohort [74]. These methods are promising precisely because they measure change over time, but they also raise additional concerns about adherence, reproducibility, privacy, and algorithmic bias (Figure 2, Table 1).

## 6. A Suggested Workflow for Clinical Formulation

**Figure 3:**
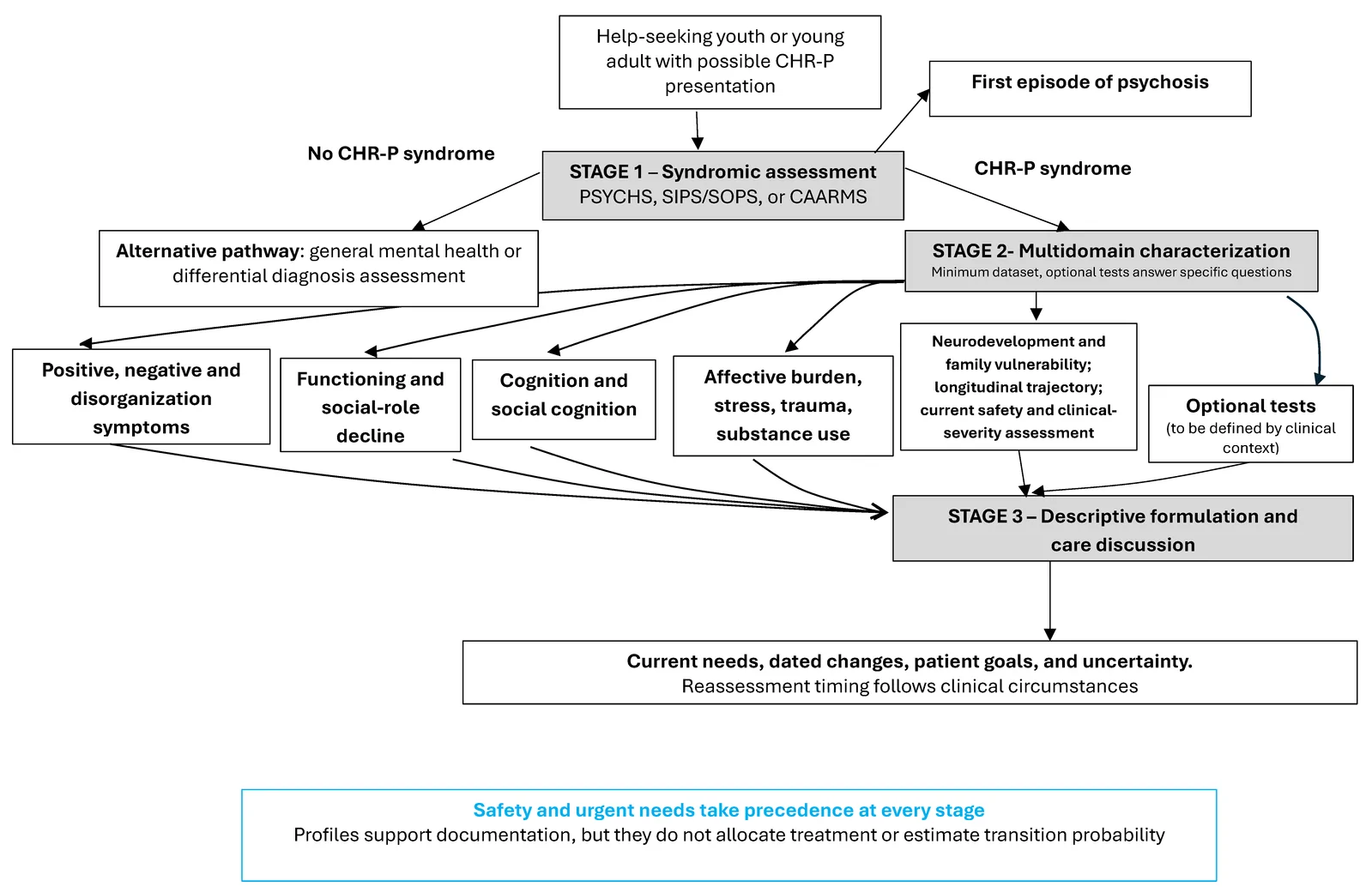
**Proposed workflow from validated CHR-P ascertainment to multidomain characterization and structured integration.** The workflow is a conceptual organization of assessment and documentation, not a validated decision algorithm. Reassessment timing depends on clinical change, safety, patient preference, and service context. CHR-P, clinical high risk for psychosis; FEP, first-episode psychosis; PSYCHS, Positive SYmptoms and Diagnostic Criteria for the CAARMS Harmonized with the SIPS; SIPS, Structured Interview for Psychosis-Risk Syndromes; SOPS, Scale of Psychosis-Risk Symptoms; CAARMS, Comprehensive Assessment of At-Risk Mental States.

Figure 3 presents the sequence of clinical assessment and formulation, whereas Figure 2 distinguishes the purpose and resource requirements of additional assessments. A trained clinician is encouraged to first evaluate a help-seeking individual using the PSYCHS, SIPS/SOPS, or CAARMS; established psychosis would redirect care to an appropriate first-episode pathway. However, in cases where CHR-P criteria are not met, assessment and treatment of other mental health needs continue in order to identify the reason for presentation to the mental health specialist. Urgent safety needs take precedence at every stage, and their management is not the objective of this article.

The second stage records symptoms, functioning, cognitive complaints, affective/stress burden, substance use, developmental and family history, safety, and longitudinal change using the minimum dataset. Optional assessments answer defined questions, while research biomarkers are recorded separately with their limitations and should not be used alone to resolve diagnostic ambiguity.

The third stage integrates these observations into a descriptive formulation. Current burden and patient-identified priorities are documented separately from observed change and any prognostic interpretation supported by independent evidence. The output records the reasons for follow-up and care, the patient’s goals, and unresolved uncertainty, without generating a transition probability.

## 7. Clinical Interpretation Profiles

The profiles in Table 2 are provisional, non-exclusive summaries of the main sources of current burden. Clinicians would first record each domain’s source measure or observation, current impact, change from the individual’s baseline, and alternative explanations. A dominant profile would be assigned only when the available history and the patient’s priorities identify a clear principal source of distress or impairment. However, when several domains are similarly prominent, multiple descriptors or a mixed profile would be recorded, and uncertainty should be retained rather than resolved by an arbitrary score. Recent syndromic worsening describes observed symptoms, not an inferred progression toward inevitable psychosis. Proposed anchors require manual, independent ratings of shared cases and prospective reliability testing before these profiles can support standardized stratification.

For operationalization, recent syndromic worsening requires a documented increase in interview-defined symptoms relative to a dated assessment or credible baseline, after considering alternative explanations, and no universal minimum change is proposed. Functional deterioration requires corroborated loss of social or role participation relative to the personal baseline, and affective/stress and developmental descriptors require evidence that those difficulties chiefly account for current needs; they do not exclude co-occurring symptom worsening. The mixed descriptor is to be applied when no defensible single priority emerges. Clinical urgency always overrides any profile and requires dedicated interventions outside the scope of this workflow. The definitions included in this conceptual model are starting points for reliability testing, not validated diagnostic boundaries.

## 8. Descriptive Visualization of the Clinical Formulation

**Figure 4:**
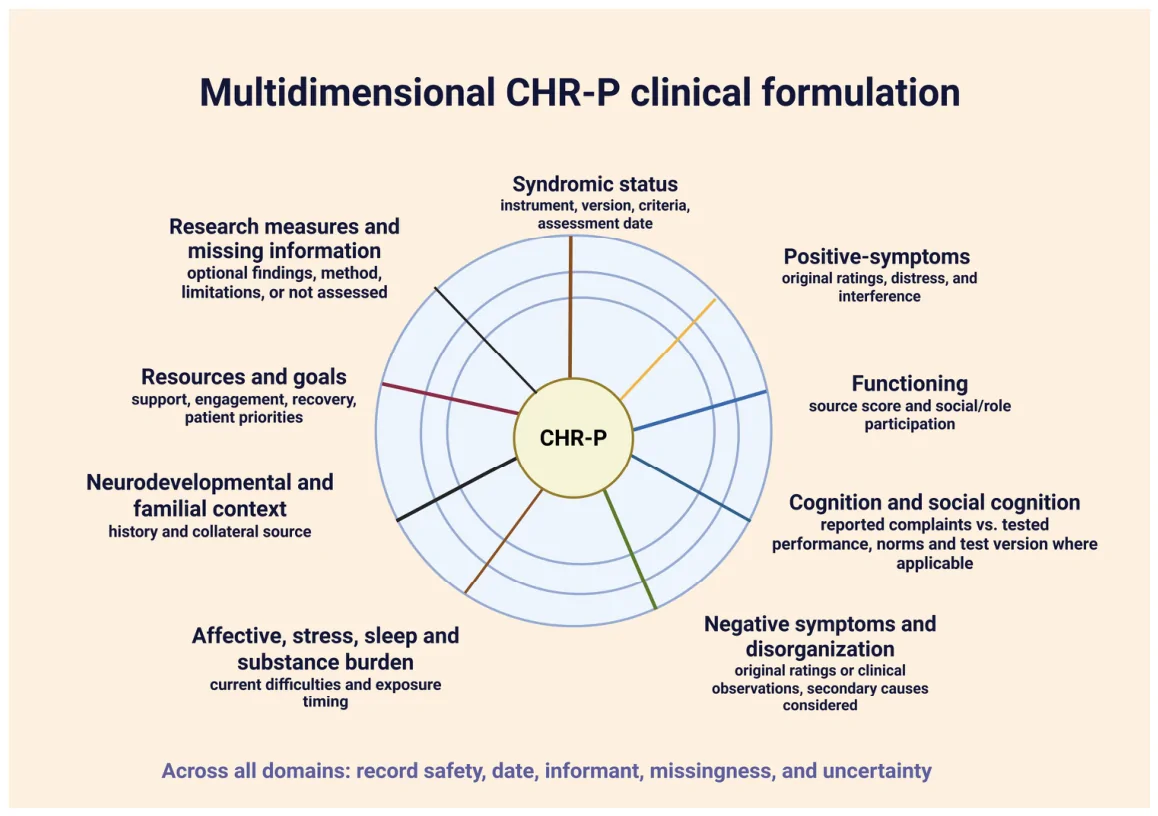
**Descriptive template for multidimensional clinical formulation.** Each row records source information, current clinical impact, and direction of change or uncertainty. Original instrument scores can be retained but are not rescaled, pooled, or assigned prognostic weights. Syndromic classification, current burden, developmental context, support, and research observations remain distinguishable. This is a documentation template whose reproducibility and usefulness require evaluation. Created in BioRender. Vasiliu, O. (2026) https://BioRender.com/00told2. (accessed on 5 September 2026).

**Figure 5:**
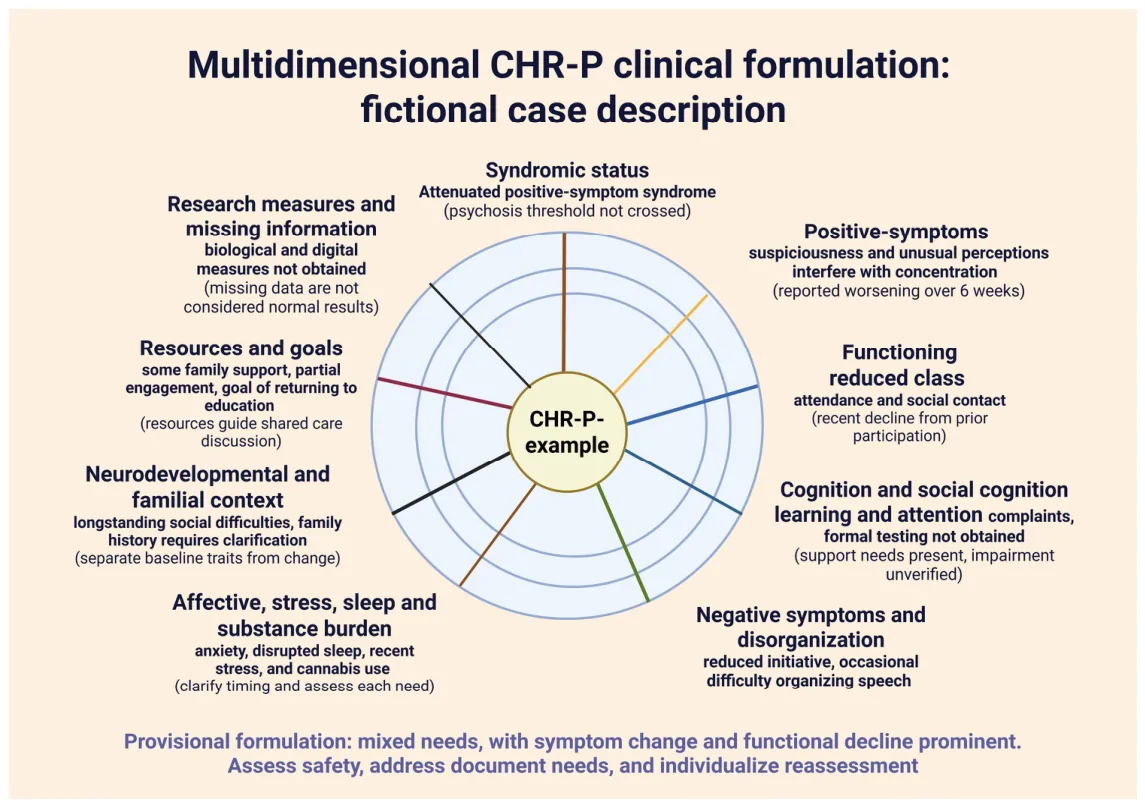
**Fictional example of descriptive multidimensional formulation.** N.B.: All case details are hypothetical. Cognitive complaints are distinguished from measured impairment, and unavailable biological or digital measures remain explicitly unassessed. Proposed care questions require ordinary clinical assessment and shared decision-making. Created in BioRender. Vasiliu, O. (2026). https://BioRender.com/8zlui5u. (accessed on 5 September 2026).

Available individualized calculators demonstrate that multivariable prediction is feasible [33], but research focused on systematic external validation shows that many published psychosis-risk models lose performance outside their derivation samples [75,76,77]. The present visualization serves a different, immediate purpose, which is making clinical observations, change, and uncertainty inspectable. Figure 4 and Figure 5 follow this principle and use descriptive matrices to organize the information in a conceptual structure. The mapping rule is to retain the original instrument and score, where measured, beside a plain-language description of its clinical impact and observed change. The assessment date, recall period, informant, and missingness must accompany interpretation, with the syndromic classification being categorical and reported separately. Formal cognitive scores require appropriate norms, while untested complaints are labeled as reported difficulties. Support, recovery, and treatment engagement are recorded descriptively and cannot subtract from symptom severity or presumed risk. Independent raters should receive the same source information and record disagreements before consensus discussion.

To illustrate how the proposed framework may be applied to individual patients, Figure 5 presents a fictional young adult who meets an attenuated positive-symptom syndrome under a structured SIPS assessment, without crossing the psychosis threshold. The most prominent signals are the recent worsening of positive symptoms, functional decline, negative symptoms and disorganization, and an unfavorable longitudinal trajectory, whereas cognitive, affective/stress-related, and neurodevelopmental or familial vulnerabilities are present only at moderate levels. Biomarkers and digital information are unavailable in this case and, consequently, they are not included. Taken together, this pattern is mostly consistent with a mixed/high-complexity profile that requires close longitudinal reassessment and a multidomain intervention within integrated case management. This example illustrates the organization of clinical information rather than providing a validated estimate of transition probability, which is a future objective for clinical studies, in case the model receives appropriate experimental validation.

## 9. Clinical Safeguards and Provisional Care Implications

Where frank psychosis is detected, the proposed framework allows for immediate superseding, and assessment continues with the appropriate FEP pathway. Also, severe suicidality, catatonia, risk of aggression, severe psychomotor agitation, disorganization, inability to care for oneself, or other markers of clinical severity require acute risk management irrespective of the multidomain profile. Potentially clinically significant investigation findings require appropriate medical follow-up, but an isolated research biomarker should not determine a CHR-P prognosis or treatment choice.

Suicide risk has particular importance in such a vulnerable population and requires flexibility in clinical care adjusted to the projected short-term evolution and complications. A recent meta-analysis (2025) that evaluated adolescents and youth with UHR (*n* = 15 studies) reported high pooled prevalences of lifetime suicidal ideation (58%), non-suicidal self-injury (37%), and suicide attempts (25%) [78]. These data support a two-track approach in which psychosis-transition assessment never replaces ordinary psychiatric safety assessment. Stigma also begins before psychosis onset; an updated systematic review (*n* = 38 studies, *N* = 2560 participants with UHR) found evidence of public, perceived, self-, associative, and stress-related stigma in UHR populations [79]. Risk communication should therefore emphasize uncertainty, the reversibility of the risk state, and the fact that CHR-P is not equivalent to the diagnosis of an impending SSD.

Reassessment frequency in individuals with CHR-P should depend on symptom changes, functional deterioration, safety, distress, family concerns, patient preferences, and service capacity, and this workflow suggests no fixed clinical schedule. NICE guidance recommends structured monitoring when symptoms, impairment, or distress persist without established psychosis, with frequency determined by clinical circumstances [80,81]. Research assessments at prespecified time points, as proposed in Section 11, must not delay clinically indicated review.

Links between the proposed profiles and care priorities are hypotheses about organizing needs assessment, not evidence that profile-based treatment improves outcomes. Antipsychotic medication should not be an automatic output of a risk score, because balancing the risks and benefits of medication is always needed. Earlier guidance was in favor of more cautious psychological first-line approaches [82], and the most recent meta-analysis (*n* = 24 studies, *N* = 3236 CHR-P individuals) in this field (2025) found no robust sustained superiority of any active preventive intervention over control conditions across transition, symptoms, and functioning [83]. That type of result enhances the case for a stratified perspective, because heterogeneous populations are unlikely to respond equally to uniform intervention strategies.

## 10. Discussion and Relationship to Existing Multidimensional Approaches

The proposed architecture in this conceptual model aligns with the direction of large-scale contemporary research, e.g., the AMP SCZ is recruiting approximately 2000 CHR-P individuals and 640 healthy controls across 43 sites in 13 countries and collects clinical, environmental, and cognitive data alongside neuroimaging, electrophysiology, biospecimens, speech/facial-expression measures, smartphone data, and actigraphy [84,85]. Importantly, its outcomes extend beyond transition to remission or persistence of CHR status, negative symptoms, mood/anxiety symptoms, and psychosocial functioning, highlighting the shift required for clinically meaningful multidimensional prediction.

The contribution proposed here is narrower: an explicit separation of present needs, observed change, and prognostic uncertainty, with a minimum dataset and a descriptive output suitable for services with different resources. This distinction has practical consequences: a clinic should be able to document current needs without acquiring research biomarkers or administering a full neurocognitive battery; optional tests require a defined question, while multimodal prognostic work requires separate model development and validation; and feasibility does not establish effectiveness, and improving documentation is not equivalent to improving functioning or preventing psychosis. Comparing the framework with usual structured assessment is therefore more informative than assuming that additional domains are beneficial.

## 11. Proposed Validation Strategy

The primary intended use of the current framework is structured needs assessment and clinical formulation in individuals with CHR-P. A first prospective pilot would evaluate the completeness of the Tier-1 dataset, time and burden, patient acceptability, and the reproducibility of domain descriptions and provisional profile assignment. A manual would specify source measures, recall periods, definitions of change, and rules for overlapping or uncertain profiles, and independent ratings would be completed before consensus adjudication. Agreement would be reported with confidence intervals using intraclass correlations for continuous source scores, weighted kappa for ordinal descriptors, and unweighted kappa and percentage agreement for nominal or multiple-profile assignments.

For a subsequent prospective cohort, the proposed primary patient outcome is role functioning at 12 months, measured continuously with the GF:R and adjusted for baseline functioning. Secondary outcomes are social functioning at 12 months (GF:S), time to psychosis transition through 24 months, CHR-P remission or persistence at 12 and 24 months, and cumulative psychiatric hospitalization days through 24 months. These outcomes are assessed regardless of transition where applicable. For this endeavor, outcomes such as quality of life, symptom burden, service use, and patient experience remain exploratory.

Repeated functioning scores would have to be analyzed with baseline-adjusted longitudinal mixed models accounting for site. Psychosis-transition analyses would address interval censoring when onset is known only between assessments and treat death as a competing event. CHR-P persistence, remission, and psychosis may be examined in a prespecified multistate model, allowing return from remission to CHR-P where observed. Hospitalization and functional impairment can coexist with any clinical state and must not be treated as mutually exclusive competing events. Missing outcomes require documented reasons, suitable imputation or joint modeling assumptions, and sensitivity analyses for informative dropout. Association and prognostic analyses cannot establish that framework-guided care causes better outcomes.

If prognostic modeling is subsequently pursued, a prespecified clinical-only model including interview findings, routine functioning, safety, comorbidity, and developmental context would be compared with additions of formal cognitive measures and then research biomarkers. Penalization, sample-size justification, internal validation, and independent external validation would be planned before analysis. TRIPOD+AI and PROBAST+AI provide relevant reporting and appraisal standards [86,87], alongside outcome-specific sample-size methods [88]. Calibration, discrimination, net benefit, and subgroup performance apply to these separate prediction models, not to the descriptive profiles. A later pragmatic comparison of framework-guided care with usual assessment would be required to test clinical utility and avoid conflating prediction with treatment benefit. The staged evaluation of the proposed formulation framework is summarized in Table 3.

## 12. Ethical and Implementation Considerations

A CHR-P classification followed by non-transition is not necessarily a diagnostic false positive: the risk state may have been correctly identified, and current needs may persist. Misclassification and prognostic false positives must be distinguished based on predefined criteria. Developmental differences, cultural context, trauma, affective illness, and substance effects should be reconsidered before interpreting unusual experiences as deterioration, and neurodivergence alone should not increase a presumed risk category or prevent access to appropriate support.

Communication should begin with the person’s current difficulties and goals. Clinicians can explain that the assessment describes experiences and their changes, that future outcomes remain uncertain, and that many people do not develop psychosis. Family involvement should follow consent, capacity, and safeguarding requirements. Written summaries should separate observations from hypotheses, avoid deterministic profile labels, and allow the patient to correct them, check individual understanding, and revisit the interpretation as circumstances change. Optional digital or genomic assessments require specific consent, data minimization, and a clear account of access, incidental findings, and the right to decline without losing care. Fairness should be evaluated across demographic groups and service settings.

Implementation should follow feasibility and reliability testing, external evaluation, and a prospective comparison with usual structured care. Existing stepped-care feasibility work demonstrates practical possibilities and barriers but does not validate these profiles or their treatment implications [89]. A later pragmatic trial would need to assess patient-centered benefits and unintended effects, including stigma, overtreatment, missed deterioration, and assessment burden, rather than documentation quality alone.

## 13. Limitations

This perspective is limited by a targeted, non-systematic evidence base and author-led synthesis. The original search is not fully reproducible from the historical description, and the documented supplementary searches improve transparency but still do not establish completeness or remove selection bias. No formal certainty grading, consensus exercise, or independent duplicate evidence selection was performed. Direct CHR-P evidence is distinguished from findings extrapolated from established psychosis, but study populations, outcome definitions, and measurement methods remain heterogeneous.

The proposed tiers, profile descriptors, mapping procedure, and profile-to-care questions have not been validated. Removing normalized coordinates prevents unwarranted numerical precision but does not itself establish inter-rater reliability or clinical benefit. Current impairment, developmental context, and prognostic variables may correlate without being interchangeable.

Biological and digital measures have not yet demonstrated incremental value, and findings from small acute-psychosis samples cannot be assumed to generalize to CHR-P. Availability and missingness may reflect service resources and participant selection; therefore, clinical practice must address these situations carefully. Research measures must therefore be evaluated against a clinical-only comparator rather than presumed to improve care.

Training requirements, assessment burden, cultural and language differences, cost, privacy, and inequitable access may limit implementation. The proposed outcomes and statistical approaches require refinement with patients, clinicians, and methodologists before prospective registration. Clinical-utility testing is needed for formulation and care planning; calibration and discrimination additionally apply only if a separate prognostic model is developed.

## 14. Conclusions

This perspective proposes a transparent way to organize established CHR-P assessment domains around current needs and observed change. A validated syndromic interview remains the entry point, while a minimum clinical dataset supports descriptive formulation and optional assessments address specific questions.

The profiles and visual templates remain provisional, and they should not determine transition probabilities, treatment allocation, or fixed monitoring schedules. Their potential clinical value lies in clearer documentation and shared discussion of care priorities and must be demonstrated through reliability, feasibility, external, and patient-outcome evaluation. Current treatment and follow-up remain guided by clinical indications, established guidance, and patient preferences, but further research is expected to provide clearer guidance toward personalized approaches for individuals with CHR-P.

## Figures and Tables

**Table 1:** Proposed minimum clinical dataset and optional structured assessments.

Domain	Minimum Information or Optional Method	Purpose	Status
**Syndromic assessment**	One trained PSYCHS, SIPS/SOPS or CAARMS assessment; record instrument/version, criteria, date, and psychosis threshold	Establish interview-defined status; distinguish established psychosis	Tier 1
**Positive symptoms**	Interview ratings; onset, frequency, distress, conviction, and recent change	Describe current impact and change	Tier 1
**Disorganization**	Relevant interview items and clinical observation	Describe current impact and change4	Tier 1
**Negative symptoms**	Interview items plus clinical history; consider secondary causes	Describe motivation, expression, and functional impact	Tier 1
**Functioning**	Current and previous social/role participation; one appropriate structured measure such as SOFAS or GF:S/GF:R	Record current impairment and change from personal baseline	Tier 1
**Cognitive difficulties**	Brief enquiry about learning, attention, memory, and effects on daily tasks	Identify support needs and whether testing is useful	Tier 1 inquiry
**Formal cognition and social cognition**	Selected age/language-appropriate tasks; BACS, MCCB, or focused emotion-recognition/theory-of-mind measures are examples	Clarify a specific difficulty; retain source scores and norms	Tier 2 optional
**Mood, anxiety, stress, trauma, sleep**	Brief clinical assessment; additional symptom scales when indicated	Document distress, differential diagnosis, and care needs	Tier 1
**Substance use and treatment exposure**	Substance timing/frequency; prescribed and non-prescribed medication; previous care	Assess attribution, interactions, and engagement	Tier 1
**Neurodevelopment and family context**	Developmental baseline, cultural context, relevant family history, and collateral information when available and consented	Distinguish longstanding differences from recent change	Tier 1
**Longitudinal trajectory and resources**	Dated change, collateral source, support, goals, recovery, and follow-up responsibility	Document priorities, uncertainty, and continuity	Tier 1
**Safety and urgent needs**	Suicidality, self-harm, self-care, safeguarding, and need for urgent assessment	Identify immediate care requirements independently of transition	Tier 1

BACS = Brief Assessment of Cognition in Schizophrenia; CAARMS = Comprehensive Assessment of At-Risk Mental States; CHR-P = Clinical High Risk for Psychosis; GF:S/GF:R = Global Functioning: Social/Global Functioning: Role; MCCB = MATRICS Consensus Cognitive Battery; PSYCHS = Positive SYmptoms and Diagnostic Criteria for the CAARMS Harmonized with the SIPS; SIPS = Structured Interview for Psychosis-Risk Syndromes; SOFAS = Social and Occupational Functioning Assessment Scale; SOPS = Scale of Psychosis-Risk Symptoms. Tier assignments are an author-proposed feasibility hierarchy, not a formal evidence grade. Additional validated scales can be used where clinically useful, and all listed instruments need not be administered, depending on the context of assessment.

**Table 2:** Provisional descriptive clinical profiles and care questions.

Provisional Descriptor	Proposed Descriptive Anchor	Care Question to Assess Individually
**Recent syndromic worsening**	Dated worsening of interview-defined positive symptoms or disorganization, after considering alternative explanations	Is earlier specialist reassessment required? Has the psychosis threshold been crossed?
**Predominant functional deterioration**	Loss of social or role participation in the main current difficulty; symptoms and cognitive complaints are recorded separately	What educational, occupational, social, or rehabilitation support is needed?
**Predominant affective or stress-related burden**	Mood, anxiety, trauma/stress, sleep, or substance-related difficulties chiefly account for current distress	Which co-occurring conditions need assessment and indicated treatment?
**Predominant neurodevelopmental or** **social-cognitive needs**	Longstanding developmental/social difficulties chiefly explain support needs; recent change is separately assessed	How should communication and support be adapted to the person’s baseline level and preferences?
**Mixed or uncertain presentations**	Several domains have similar prominence, or attribution is unresolved; biomarkers are not required	Is multidisciplinary review useful, and which needs and uncertainties should be prioritized together?

**Table 3:** Staged evaluation of the proposed formulation framework.

Phase Number	Purpose	Proposed Evaluation	Decision Before Proceeding
**1**	Operationalization	Patient/clinician input; manual; source measures; profile rules; outcome definitions	Freeze and register the protocol
**2**	Feasibility and reliability	Independent ratings; confidence intervals for agreement; completion, burden, missingness, acceptability	Revise unreliable or burdensome elements
**3**	Prospective cohort	Primary: GF:R at 12 months; secondary: GF:S at 12 months, transition to 24 months, CHR-P status at 12/24 months, hospitalization days to 24 months	Evaluate usefulness across settings and groups
**4**	Separate prognostic studies if justified	Clinical-only comparator; cognitive/biomarker additions; internal and independent external validation	Demonstrate calibration, discrimination, and net benefit
**5**	Clinical-utility evaluation	Pragmatic comparison with usual structured care; functioning, experience, harms, resources, and equity	Demonstrate patient benefit before recommending profile-based care

## Data Availability

No new data were created or analyzed in this study. Data sharing is not applicable to this article.

## Abbreviations

The following abbreviations are used in this manuscript:

AI	Artificial intelligence
AMP SCZ	Accelerating Medicines Partnership Schizophrenia
APS	Attenuated psychosis syndrome
ARMS	At-risk mental state
BACS	Brief Assessment of Cognition in Schizophrenia
BLIPS	Brief limited intermittent psychotic symptoms
CAARMS	Comprehensive Assessment of At-Risk Mental States
CHR	Clinical high risk
CHR-P	Clinical high risk for psychosis
CSF	Cerebrospinal fluid
DSM-III-R	Diagnostic and Statistical Manual of Mental Disorders, Third Edition, Revised
DSM-IV	Diagnostic and Statistical Manual of Mental Disorders, Fourth Edition
DSM-5	Diagnostic and Statistical Manual of Mental Disorders, Fifth Edition
DSM-5-TR	Diagnostic and Statistical Manual of Mental Disorders, Fifth Edition, Text Revision
DTI-ALPS	Diffusion tensor imaging along the perivascular space
EEG	Electroencephalography
EPA	European Psychiatric Association
FEP	First-episode psychosis
GABA	Gamma-aminobutyric acid
GF:R	Global Functioning: Role
GF:S	Global Functioning: Social
IL-6	Interleukin-6
MATRICS	Measurement and Treatment Research to Improve Cognition in Schizophrenia
MCCB	MATRICS Consensus Cognitive Battery
MMN	Mismatch negativity
MRI	Magnetic resonance imaging
MRS	Magnetic resonance spectroscopy
NAPLS	North American Prodrome Longitudinal Study
NICE	National Institute for Health and Care Excellence
OASIS	Outreach and Support in South London
P300	P300 event-related potential
PRISMA	Preferred Reporting Items for Systematic Reviews and Meta-Analyses
PROBAST+AI	Prediction model Risk Of Bias Assessment Tool plus Artificial Intelligence
PRS	Polygenic risk score
PSYCHS	Positive SYmptoms and Diagnostic Criteria for the CAARMS Harmonized with the SIPS
SANS	Scale for the Assessment of Negative Symptoms
SIPS	Structured Interview for Psychosis-Risk Syndromes
SOFAS	Social and Occupational Functioning Assessment Scale
SOPS	Scale of Psychosis-Risk Symptoms
SPI	Schizophrenia Proneness Instrument
SPI-A	Schizophrenia Proneness Instrument, Adult version
SPI-CY	Schizophrenia Proneness Instrument, Child and Youth version
SSD	Schizophrenia spectrum disorders
ToM	Theory of mind
TRIPOD+AI	Transparent Reporting of a multivariable prediction model for Individual Prognosis Or Diagnosis plus Artificial Intelligence
UHR	Ultra-high risk
UHR-P	Ultra-high risk for psychosis
US	United States

## Appendix A. Supplementary Searches Performed During Revision

Database: PubMed, queried through the NCBI E-utilities interface on 27 September 2026. These supplementary searches checked recent evidence and reviewer-raised topics while retaining 31 August 2026 as the publication-date cutoff. They are not the historical queries used to prepare the original manuscript.

Q1: 147 records retrieved

((“clinical high risk”[Title/Abstract] OR “ultra high risk”[Title/Abstract] OR “at risk mental state”[Title/Abstract]) AND psychosis[Title/Abstract]) AND (“meta-analysis”[Publication Type] OR “systematic review”[Publication Type] OR PSYCHS[Title/Abstract] OR “AMP SCZ”[Title/Abstract] OR OASIS1000[Title/Abstract]) AND (“2021/01/01”[Date—Publication]: “2026/08/31”[Date—Publication])

Q2: 30 records retrieved

(psychosis[Title/Abstract] OR schizophrenia[Title/Abstract]) AND (glymphatic[Title/Abstract] OR “DTI-ALPS”[Title/Abstract]) AND (“1900/01/01”[Date—Publication]: “2026/08/31”[Date—Publication])

Q3: 20 records retrieved

(“TRIPOD+AI”[Title] OR “PROBAST+AI”[Title]) AND (“2024/01/01”[Date—Publication]: “2026/08/31”[Date—Publication])

Selection and interpretation

The 197 retrieved records across the three queries were inspected at the title level. Abstracts and selected full texts were considered purposively for their relevance to syndromic assessment, current burden, transition or functional outcomes, feasibility, and the specific issues raised in the review. The broad queries also retrieved tangential records; these did not supply claims in the revised framework. Evidence syntheses and direct CHR-P studies were prioritized over isolated associations in established psychosis. The suggested acute-psychosis glymphatic study was retained as indirect, hypothesis-generating evidence and considered alongside direct CHR-P findings. Foundational references and official clinical guidance were checked separately. No formal included-study count, exhaustive eligibility process, or independent duplicate screening is claimed.
